# SLC25A35 carrier endows mitochondrial phosphoenolpyruvate with glyceroneogenesis

**DOI:** 10.1093/lifemeta/loag016

**Published:** 2026-06-10

**Authors:** Xinyu Liu, Wei Meng, Xun Huang

**Affiliations:** State Key Laboratory of Metabolism and Regulation in Complex Organisms, Taikang Center for Life and Medical Sciences, School of Basic Medical Sciences, Wuhan University, Wuhan, Hubei 430071, China; Institute of Genetics and Developmental Biology, Chinese Academy of Sciences, Beijing 100101, China; State Key Laboratory of Metabolism and Regulation in Complex Organisms, Taikang Center for Life and Medical Sciences, School of Basic Medical Sciences, Wuhan University, Wuhan, Hubei 430071, China; Institute of Genetics and Developmental Biology, Chinese Academy of Sciences, Beijing 100101, China; State Key Laboratory of Metabolism and Regulation in Complex Organisms, Taikang Center for Life and Medical Sciences, School of Basic Medical Sciences, Wuhan University, Wuhan, Hubei 430071, China; Institute of Genetics and Developmental Biology, Chinese Academy of Sciences, Beijing 100101, China

**Mitochondria not only synthesize ATP as cellular energy factories but also serve as core metabolic hubs that supply precursors for specialized biosynthesis. In a recent study published in *Cell*, Yamamuro *et al.* [1] identify solute carrier family 25 member 35 (SLC25A35) as the elusive mitochondrial phosphoenolpyruvate (PEP) carrier. This mitochondrial PEP export supports glycerolipid production and regulates hepatic and adipose glyceroneogenesis, connecting mitochondrial meta­bolism to triglyceride formation and fatty liver development.** 

Lipid storage and mobilization are essential processes of systemic energy homeostasis. In the adipose tissue and the liver, fatty acids are esterified and incorporated into triglycerides (TGs) for storage and subsequently hydrolyzed from TGs and oxidized during periods of increased energy demand. This dynamic process requires not only fatty acids but also a constant supply of glycerol-3-phosphate (G3P), which serves as the glycerol backbone of TGs and phospholipids. Although considerable attention has been devoted to pathways controlling fatty acid synthesis and oxidation, the mechanisms that regulate glycerol backbone production have received comparatively less attention.

PEP occupies a key position in intermediary metabolism. Owing to its high-energy phosphate bond, PEP participates in diverse metabolic pathways. In lipogenic tissues, PEP can be converted into glyceraldehyde-3-phosphate (GA3P) and dihydroxyacetone phosphate (DHAP), which subsequently generate G3P, the glycerol backbone required for glycerolipid synthesis. PEP is synthesized from oxaloacetate (OAA) by phosphoenolpyruvate carboxykinase (PEPCK), an enzyme first purified and characte­rized > 70 years ago [2]. Early studies further demonstrated that PEP metabolism is compartmentalized between cytosolic and mitochondrial fractions [3], laying the foundation for the later identification of two mammalian PEPCK isoforms: the cytosolic phosphoenolpyruvate carboxykinase 1 (PCK1) and the mitochondrial phosphoenolpyruvate carboxykinase 2 (PCK2). Historically, PCK1 has received more attention because of its well-established role in hepatic gluconeogenesis, leading to the widespread view that physiologically relevant PEP is primarily generated in the cytosol. However, accumulating evidence has highlighted pro­minent functions of PCK2 in mitochondrial carbon metabolism. Studies in mouse liver demonstrated that mitochondrial PCK2 contributes to gluconeogenic flux yet cannot fully substitute for the loss of *Pck1* [4], suggesting functional differences between mitochondria- and cytosolic-derived PEP pools. Subsequent work further established a role for PCK2 in regulating hepatic glucose production and metabolic adaptation [5]. Moreover, isotope tracing studies in nutrient-stressed cancer cells revealed that PCK2-derived carbon can be incorporated into the glycerol backbone of phospholipids [6], implicating mitochondrial PEP metabolism in lipid biosynthesis beyond its classical role in gluconeogenesis. Despite these observations, it has remained unclear how mitochondria-derived PEP reaches the cytosol and whether mitochondrial PEP possesses metabolic functions distinct from those of cytosolic PEP.

To investigate the origin of PEP in lipogenic cells, Yamamuro *et al.* performed isotope tracing studies using ^13^C-labeled pyruvate in isolated mitochondria. Remarkably, the mitochondria derived from white and brown adipocytes predominantly gene­rated M + 3 PEP through a pyruvate-to-PEP bypass pathway, indicating direct conversion of pyruvate to OAA and subsequently to PEP. In contrast, the mitochondria from human embryonic kidney 293 T (HEK293T) cells and myoblasts generated substantially lower amounts of M + 3 PEP, suggesting that mitochondrial PEP production is highly cell-type–specific. Consistent with these observations, transcriptional analyses revealed that *Pck2* is the dominantly expressed PEPCK in adipocytes, whereas *Pck1* expression remains relatively low.

Functional studies further established the importance of PCK2-dependent mitochondrial PEP production. Deletion of *Pck2* significantly reduced incorporation of pyruvate-derived carbon into the glycerol backbone of glycerolipids, demonstrating that mitochondrial PEP contributes directly to glyceroneoge­nesis. These findings raised an important mechanistic question. Because the inner mitochondrial membrane is impermeable to charged metabolites, mitochondrial PEP would require a dedicated transporter to reach the cytosol. Yet no such transporter had been identified despite decades of investigation into mitochondrial metabolism.

To address this, the authors integrated multiple analyses to identify lipogenesis-associated mitochondrial carrier proteins. Among the candidates, solute carrier family 25 member 35 (SLC25A35) emerged as a particularly intriguing molecule. Although belonging to the SLC25 family of mitochondrial transporters, SLC25A35 had remained an orphan carrier with no clearly defined physiological substrate. Importantly, *Slc25a35* expression was enriched in adipocytes and positively correlated with lipogenic genes such as *Acaca* and *Fasn*. Its expression was also induced under conditions associated with enhanced lipid synthesis, further suggesting a role in anabolic metabolism.

The authors then directly examined mitochondrial PEP transport by SLC25A35. Using isolated mitochondria incubated with labeled pyruvate, they developed an assay capable of monitoring mitochondrial PEP export. Control mitochondria actively released newly synthesized PEP into the surrounding medium, whereas *Slc25a35*-deficient mitochondria displayed a profound defect in PEP export. These findings provided compelling evidence that SLC25A35 is required for the movement of PEP across the inner mitochondrial membrane.

One of the most technically compelling aspects of the study is the biochemical reconstitution of SLC25A35 transport activity. Purified SLC25A35 was incorporated into artificial liposomes, enabling substrate transport to be examined in a cell-free system. In this setting, SLC25A35 alone was sufficient to mediate PEP transport, establishing it as a *bona fide* mitochondrial PEP transporter. Competition assays further identified PEP as a preferred substrate, whereas only weak competition was observed with several tricarboxylic acid cycle intermediates. Moreover, transport activity was enhanced under conditions favoring proton gradients, consistent with a role for electrochemical forces in driving PEP translocation across the inner mitochondrial membrane.

To complement the biochemical studies, the authors employed structural modeling and molecular dynamics simulations. AlphaFold-based structural predictions revealed a characteristic carrier-like architecture containing a central transport cavity. Computational docking identified multiple residues potentially involved in PEP recognition and binding. Mutational analyses supported these predictions, confirming that specific amino acids within the transport channel contribute to substrate transport.

**Figure 1:**
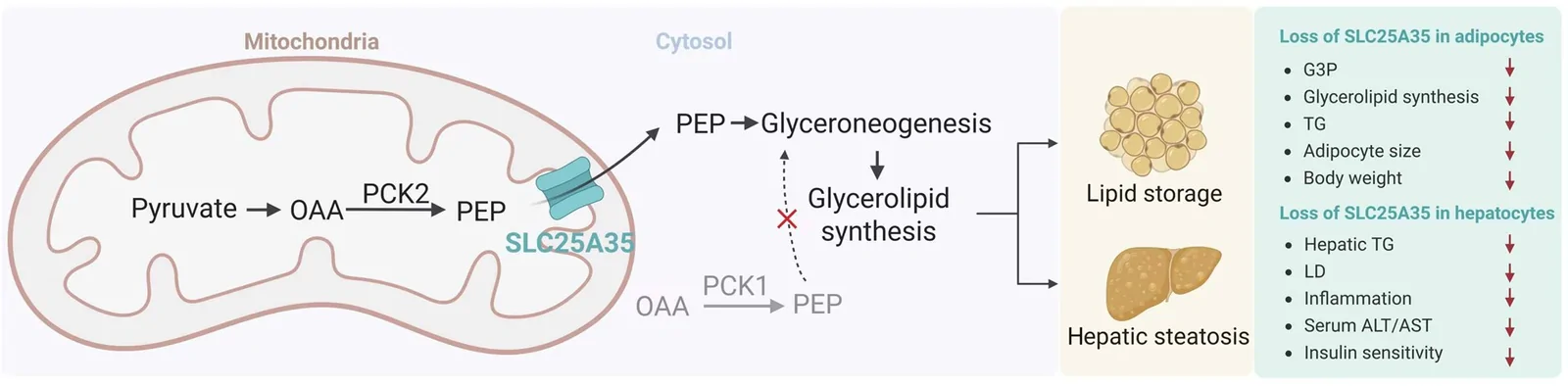
PEP is synthesized from OAA by PCK2 in the mitochondria and subsequently exported to the cytosol via SLC25A35, where it participates in cytosolic glyceroneogenesis and glycerolipid synthesis. This process promotes lipid storage in adipose tissues and hepatic steatosis. Cytosolic PEP derived from PCK1 does not contribute to glyceroneogenesis. Inhibition of *Slc25a35* attenuates glyceroneogenesis and the associated phenotypes in both the adipose tissues and the liver. Created in BioRender.

Having established SLC25A35 as a mitochondrial PEP transporter, the authors next investigated its metabolic consequences. Stable-isotope tracing demonstrated that loss of SLC25A35 dramatically reduced the production of labeled GA3P/DHAP and lowered cellular G3P levels. As a consequence, TG synthesis was markedly impaired. Importantly, further experiments revealed that the source of PEP mattered. Overexpression of the cytosolic enzyme PCK1 failed to rescue TG synthesis in *Slc25a35*-deficient adipocytes, indicating that simply increasing total cellular PEP is insufficient. Instead, glyceroneogenesis depended specifically on mitochondria-derived PEP exported through SLC25A35. Additional experiments showed that simultaneous disruption of *Pck2* and *Slc25a35* did not further suppress glyceroneogenesis beyond either manipulation alone, demonstrating that both ­proteins function within the same metabolic pathway (Fig. 1).

The physiological significance of this pathway was explored using adipose tissue-specific *Slc25a35*-knockout mice. When challenged with a high-fat diet under thermoneutral conditions, these mice displayed reduced adipose tissue mass, smaller adipocytes, and attenuated body weight gain. Notably, these phenotypes occurred without significant alterations in food intake, locomotor activity, or energy expenditure. Therefore, the reduced adiposity cannot be explained by increased energy consumption but instead reflects impaired lipid storage capacity. Consistent with this interpretation, adipose tissues from knockout mice exhibited reduced incorporation of glucose-derived carbon into the glycerol backbone of glycerolipids. These findings provide *in vivo* evidence that mitochondrial PEP export is required for efficient glyceroneo­genesis and TG accumulation in adipose tissue.

The physiological relevance of the SLC25A35–PEP pathway extends beyond adipose tissue. Analysis of human meta­bolic dysfunction-associated steatotic liver disease (MASLD) samples and obese mouse livers revealed elevated *Slc25a35* expression, implicating it in hepatic lipid accumulation. Indeed, hepatocyte-specific deletion of *Slc25a35* resulted in mitochondrial PEP accumulation and markedly reduced hepatic TG content and lipid droplet deposition. Importantly, liver-specific blockade of *Slc25a35* also attenuated hepatic inflammation and improved systemic glucose homeostasis, while acute depletion of *Slc25a35* in obese mice similarly alleviated steatosis and liver injury. These findings identify mitochondrial PEP export as a previously unre­cognized contributor to hepatic lipid accumulation and implicate SLC25A35 as a therapeutic target for MASLD.

This study goes beyond identifying a previously orphaned mitochondrial carrier, joining recent de-orphanization efforts involving the methylated amino acid carrier SLC25A45 [7, 8]. Importantly, it provides a conceptual advance in our understanding of metabolic compartmentalization, an emerging framework that emphasizes how the spatial organization of metabolites and metabolic pathways shapes cellular physiology and meta­bolic output [9, 10]. Within this framework, Yamamuro *et al.* demonstrate that mitochondria-derived PEP is not simply interchangeable with cytosolic PEP but instead constitutes a spatially distinct metabolic pool that supports glyceroneogenesis through SLC25A35-mediated export. For decades, mitochondrial contributions to lipogenesis have been largely interpreted through citrate export, which supplies cytosolic acetyl-CoA for fatty acid synthesis. By contrast, the glycerol backbone of TGs has generally been viewed as a downstream product of cytosolic carbohydrate metabolism. The discovery of the SLC25A35-dependent PEP shuttle therefore expands the role of mitochondria in lipid metabolism from supplying fatty acid precursors to coordinating both major building blocks required for glycerolipid synthesis.

From a translational perspective, the study is equally compelling. Liver-specific inhibition of *Slc25a35* ameliorates steatosis, reduces inflammation, and improves metabolic parameters in obese mice, highlighting mitochondrial PEP export as a druggable node in MASLD pathogenesis. As effective treatments for MASLD remain limited, targeting the mitochondrial PEP shuttle may represent a promising strategy for selectively restricting glycerolipid synthesis without broadly disrupting central carbon metabolism. Future studies aimed at developing pharmacological inhibitors of SLC25A35 will therefore be of considerable interest.

Several questions remain unresolved. First, although the study convincingly establishes the importance of SLC25A35 in the adipose tissue and the liver, its physiological role in other *Pck2*-expressing tissues remains unclear. Mitochondrial PEP metabolism has been implicated in pancreatic β-cell function, immune-cell activation, and cancer metabolism, raising the possibility that SLC25A35-mediated transport participates in a broader spectrum of metabolic adaptations. Second, the molecular mechanism of transport requires further investigation. High-resolution structural characterization of SLC25A35 in multiple conformational states will be necessary to define the transport cycle and substrate selectivity in greater detail. Third, it remains unknown whether SLC25A35 activity is dynamically regulated by hormonal or nutritional cues. Given the observed dependence on membrane potential, SLC25A35 may function as a metabolic ­checkpoint ­linking mitochondrial energetic status to anabolic flux.

In summary, Yamamuro *et al.* identify SLC25A35 as the mitochondrial PEP exporter that couples mitochondrial metabolism to cytosolic glyceroneogenesis and glycerolipid synthesis. Through an elegant combination of isotope tracing, metabolomics, biochemical reconstitution, structural modeling, and mouse genetics, the authors establish a mitochondrial PEP shuttle that governs lipid storage in the adipose tissue and the liver. By revealing how mitochondria directly supply a critical precursor for glycerol backbone synthesis, this study uncovers a previously unappreciated layer of metabolic regulation and identifies SLC25A35 as a promising therapeutic target for hepatic steatosis and related metabolic disorders.
